# Gait training with a wearable curara® robot for cerebellar ataxia: a single-arm study

**DOI:** 10.1186/s12938-021-00929-w

**Published:** 2021-09-08

**Authors:** Akira Matsushima, Yoichi Maruyama, Noriaki Mizukami, Mikio Tetsuya, Minoru Hashimoto, Kunihiro Yoshida

**Affiliations:** 1 Department of Neurology, JA Nagano Koseiren Kakeyu-Misayama Rehabilitation Center Kakeyu Hospital, Ueda, Japan; 2 Department of Rehabilitation, JA Nagano Koseiren Kakeyu-Misayama Rehabilitation Center Kakeyu Hospital, Ueda, Japan; 3 Department of Information Technology, International Professional University of Technology in Tokyo, Tokyo, Japan; 4 AssistMotion Inc., Ueda, Japan; 5 Faculty of Textile Science and Technology, Shinshu University, Ueda, Japan; 6 Division of Neurogenetics, Department of Brain Disease Research, Shinshu University School of Medicine, Matsumoto, Japan

**Keywords:** Spinocerebellar ataxia, Rehabilitation, Robot-assisted gait training, Wearable robot

## Abstract

**Background:**

Ataxic gait is one of the most common and disabling symptoms in people with degenerative cerebellar ataxia. Intensive and well-coordinated inpatient rehabilitation improves ataxic gait. In addition to therapist-assisted gait training, robot-assisted gait training has been used for several neurological disorders; however, only a small number of trials have been conducted for degenerative cerebellar ataxia. We aimed to validate the rehabilitative effects of a wearable “curara®” robot developed in a single-arm study of people with degenerative cerebellar ataxia.

**Methods:**

Twenty participants with spinocerebellar ataxia or multiple system atrophy with predominant cerebellar ataxia were enrolled. The clinical trial duration was 15 days. We used a curara® type 4 wearable robot for gait training. We measured the following items at days 0, 7, and 14: Scale for the Assessment and Rating of Ataxia, 10-m walking time (10 mWT), 6-min walking distance (6 mWD), and timed up and go test. Gait parameters (i.e., stride duration and length, standard deviation of stride duration and length, cadence, ratio of the stance and swing phases, minimum and maximum knee joint angles, and minimum and maximum hip joint angles) were obtained using a RehaGait®. On days 1–6 and 8–13, the participants were instructed to conduct gait training for 30 ± 5 min with curara®. We calculated the improvement rate as the difference of values between days 14 and 0 divided by the value on day 0. Differences in the gait parameters were analyzed using a generalized linear mixed model with Bonferroni’s correction.

**Results:**

Data from 18 participants were analyzed. The mean improvement rate of the 10 mWT and 6 mWD was 19.0% and 29.0%, respectively. All gait parameters, except the standard deviation of stride duration and length, improved on day 14.

**Conclusions:**

Two-week RAGT with curara® has rehabilitative effects on gait function comparable to those of therapist-assisted training. Although the long-term effects after a month of RAGT with curara® are unclear, curara® is an effective tool for gait training of people with degenerative ataxia.

*Trial registration* jRCT, jRCTs032180164. Registered: 27 February 2019; retrospectively registered. https://jrct.niph.go.jp/en-latest-detail/jRCTs032180164.

## Background

Spinocerebellar ataxia (SCA) and multiple system atrophy with predominant cerebellar ataxia (MSA-C) are neurodegenerative diseases that predominantly affect the cerebellum [1]. There is no curative pharmacological therapy for SCA or MSA-C; however, well-organized rehabilitation programs can temporarily improve ataxia [2–4]. People with degenerative ataxia are commonly treated with 2- to 6-week inpatient or home-based rehabilitation [6–8], but only a few studies on this topic meet the definition of Evidence Level II, in accordance with the Australian National Health and Medical Research Council Evidence Hierarchy [3]. This is mainly because the prevalence of degenerative ataxia is much less than that of stroke. Moreover, the optimal timing, frequency, and duration of rehabilitation for degenerative ataxia is very difficult to determine. Despite the effectiveness of inpatient rehabilitation, continuous, home-based rehabilitation is required to maintain physical function because the gains achieved by rehabilitation gradually attenuate [5, 6]. This is a clinically and socially burdensome issue for rehabilitation of degenerative ataxia.

Robot-assisted gait training (RAGT) is one of the most popular applications of robotics to rehabilitation [9–15]. RAGT has been used for various diseases, including stroke [11, 12, 14], Parkinson’s disease [13], spinal cord injury [10], and cerebral palsy [9]. However, only a few trials of RAGT have been conducted in patients with SCA or MSA-C. We developed a wearable “curara®” robot to assist in movements of the elderly or disabled people. Furthermore, we evaluated the assist conditions and immediate effects of this robot on the gait of people with stroke or cerebellar ataxia [16, 17]. We observed that several gait parameters temporarily improved with curara® in people with stroke or cerebellar ataxia under certain assist conditions. In particular, people with cerebellar ataxia benefited from the assist condition of increased range of motion at the hip and knee joints and shortened gait cycle [16, 17].

However, the temporal effects on gait function while wearing the robot do not confirm an effect on rehabilitation. Our goal was to develop a wearable robot to facilitate daily, home-based gait training for people with neurological disorders. For this purpose, this study aimed to evaluate the effects of curara® on the rehabilitation of people with cerebellar ataxia. If a positive effect of curara® is identified, this robot may be used for home-based, long-term rehabilitation of patients with degenerative ataxia.

## Results

Twenty participants were included in this study; however, one [56-year-old male with SCA6, Berg Balance Scale (BBS) score of 36, and Scale for the Assessment and Rating of Ataxia (SARA) score of 17.5 at baseline] withdrew from the study on day 3 because he no longer wished to continue participating in the trial. The remaining 19 participants completed the program without any harmful events. However, one participant (84-year-old female with SCA31, BBS score of 27, and SARA score of 17.5 at baseline) was excluded from the analysis due to extreme outliers in most of the measured items; therefore, the data from 18 participants (SCA: 13; MSA-C: 5) were analyzed. Table 1 summarizes the detailed information of the participants and results of the SARA, BBS, and timed up and go (TUG) tests. At baseline, the participants were aged 62.8 ± 9.8 years [mean ± standard deviation (SD)]; the BBS and SARA scores were 41.0 ± 6.7 and 10.6 ± 3.9, respectively. There were no differences in the SARA and BBS scores, but the TUG test score improved on day 14 (*p* = 0.002).

**Table 1:** Participant characteristics and results of the SARA, BBS, and TUG tests

No	Disease type	Age (y)/sex	SARA scores		BBS scores		TUG tests	
			Day 0	Day 14	Day 0	Day 14	Day 0	Day 14
1	SCA6	45/M	14.0	12.5	30	34	21.4	24.5
2	IDCA	77/M	9.5	7.5	44	53	18.6	11.0
3	MSA-C	65/F	5.0	3.5	51	53	12.6	10.2
4	IDCA	56/F	8.0	8.0	45	45	15.1	13.8
5	ADCA	57/F	7.5	7.0	49	51	6.8	8.3
6	SCA6	51/F	8.0	8.0	39	44	14.5	13.4
7	MSA-C	48/M	13.0	12.0	35	36	28.4	19.0
8	SCA6	61/F	13.5	12.0	35	40	25.1	22.7
9	MSA-C	72/F	13.5	11.5	33	39	26.5	20.6
10	SCA36	57/M	14.5	14.5	45	44	34.1	25.1
11	MSA-C	74/F	10.5	14.0	36	41	16.5	16.4
12	SCA31	72/M	19.5	21.0	34	19	43.1	32.4
13	SCA31	63/M	9.0	8.5	49	52	13.2	11.4
14	SCA2	50/F	11.0	11.0	37	40	14.1	12.3
15	IDCA	69/M	14.0	14.5	42	43	17.8	14.5
16	ADCA	69/F	2.0	2.0	53	54	10.2	8.9
17	ADCA	75/F	9.0	13.0	45	50	18.9	16.1
18	MSA-C	69/F	10.0	9.0	36	37	19.3	17.1
Mean ± SD			10.6 ± 4.0	10.5 ± 4.4	41.0 ± 6.9	43.1 ± 8.7	19.8 ± 8.9*	16.5 ± 6.5*

*ADCA* autosomal dominant cerebellar ataxia without genetic testing, *BBS* Berg Balance Scale, *IDCA* idiopathic cerebellar ataxia, *MSA-C* multiple system atrophy with predominant cerebellar ataxia, *SARA* Scale for the Assessment and Rating of Ataxia, *SCA* spinocerebellar ataxia, *SD* standard deviation, *TUG* timed up and go

*Statistically significant difference

**Fig. 1:**
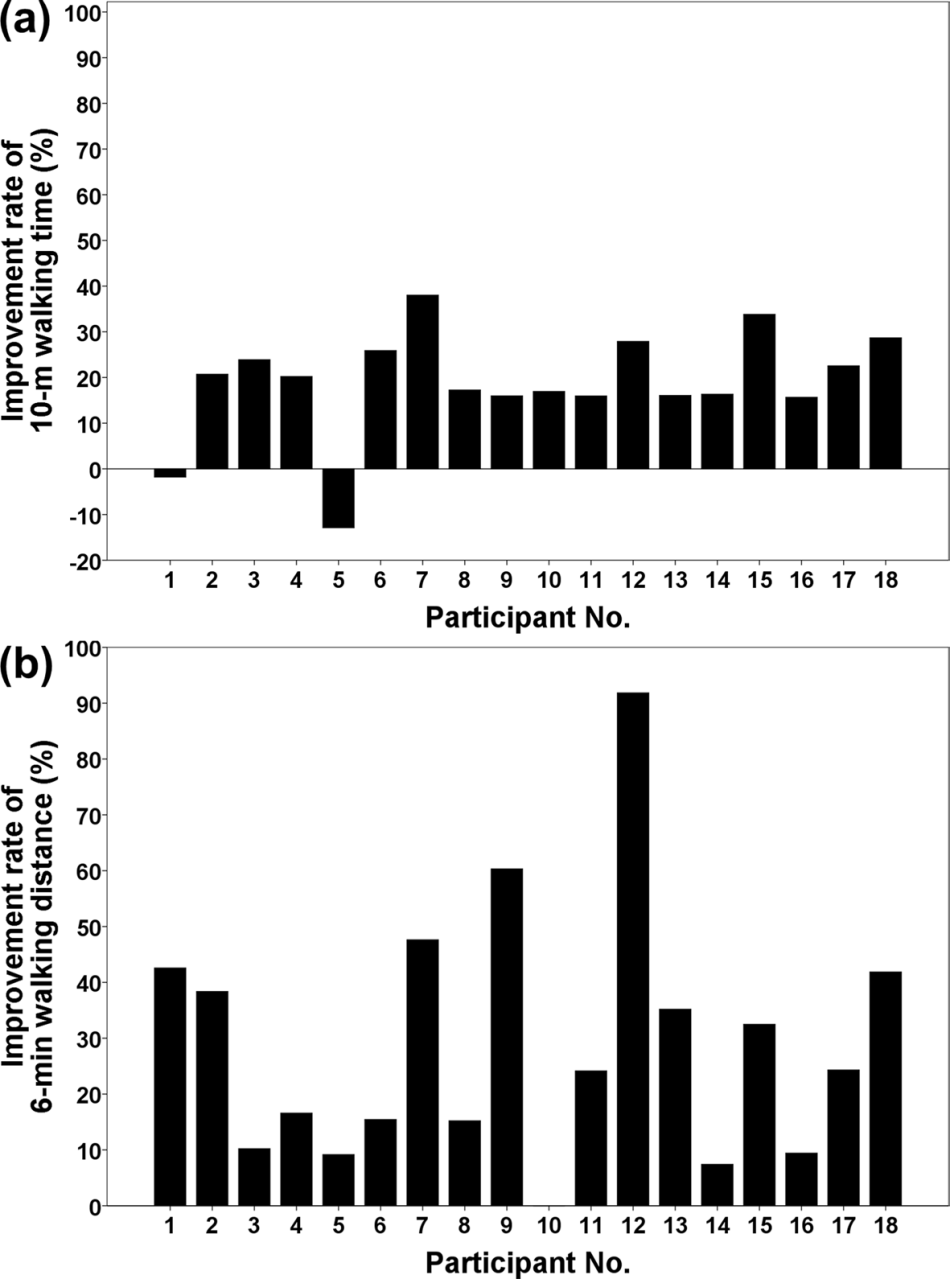
Results of the main outcome measures. **a** The improvement rate of the 10-m walking time. **b** The improvement rate of the 6-min walking distance

**Fig. 2:**
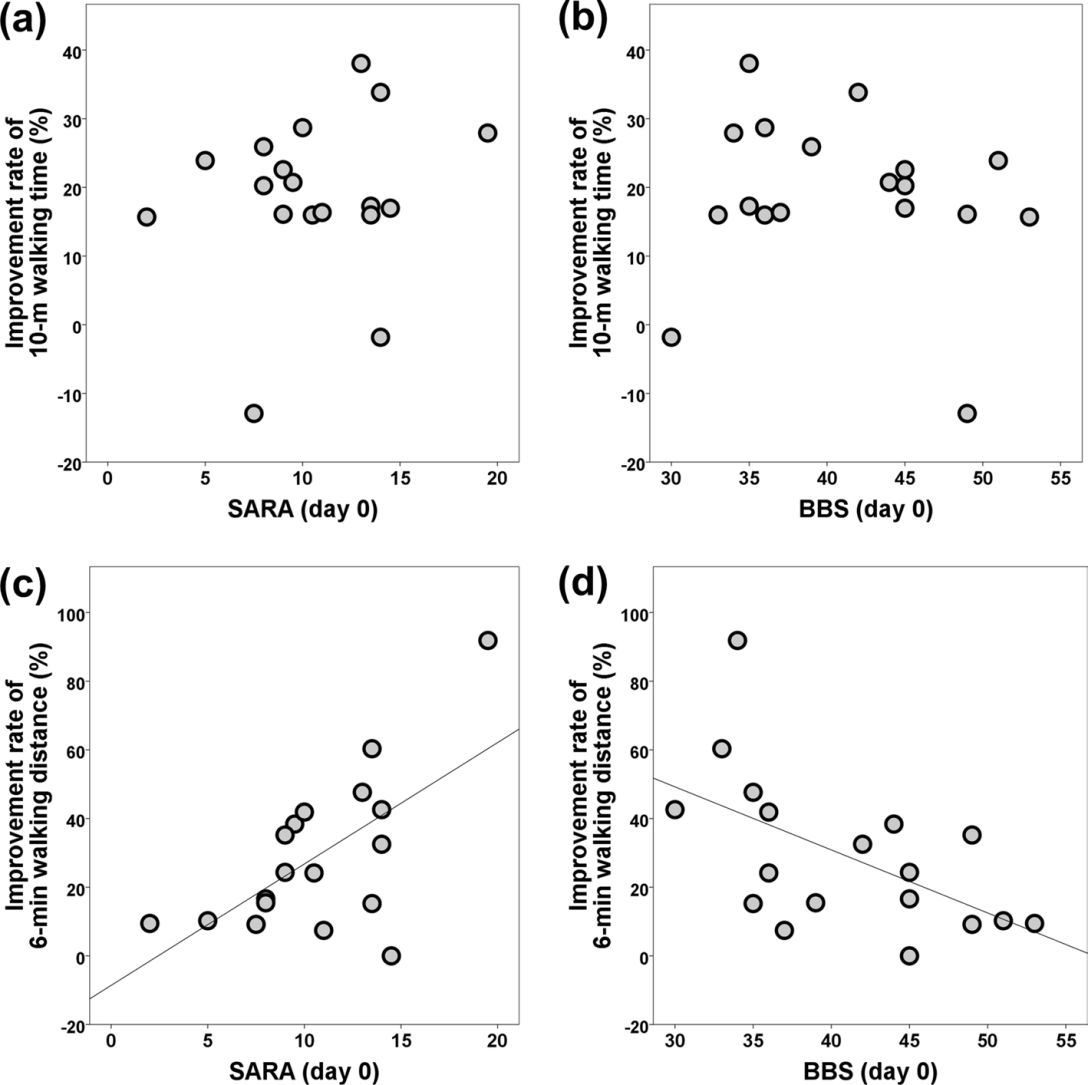
Correlation of the SARA (**a**, **c**) and BBS (**b**, **d**) scores with the main outcome measures. The improvement rate of the 6-min walking distance, but not that of the 10-m walking time, correlated with the SARA and BBS scores at baseline (day 0). *BBS* Berg Balance Scale, *SARA* Scale for the Assessment and Rating of Ataxia

Figure 1 depicts the results of the main outcome measures. Mean improvement rates for the 10-m walking time (10 mWT) and 6-min walking distance (6 mWD) were 19.0% and 29.0%, respectively. Both rates showed considerable variability between individuals, and did not correlate with each other (correlation coefficient: 0.340; *p* = 0.168). The improvement rate for the 6 mWD, but not the 10 mWT, significantly correlated with the SARA scores (correlation coefficient: 0.626) and BBS scores (correlation coefficient: − 0.557) at baseline (Fig. 2).

**Fig. 3:**
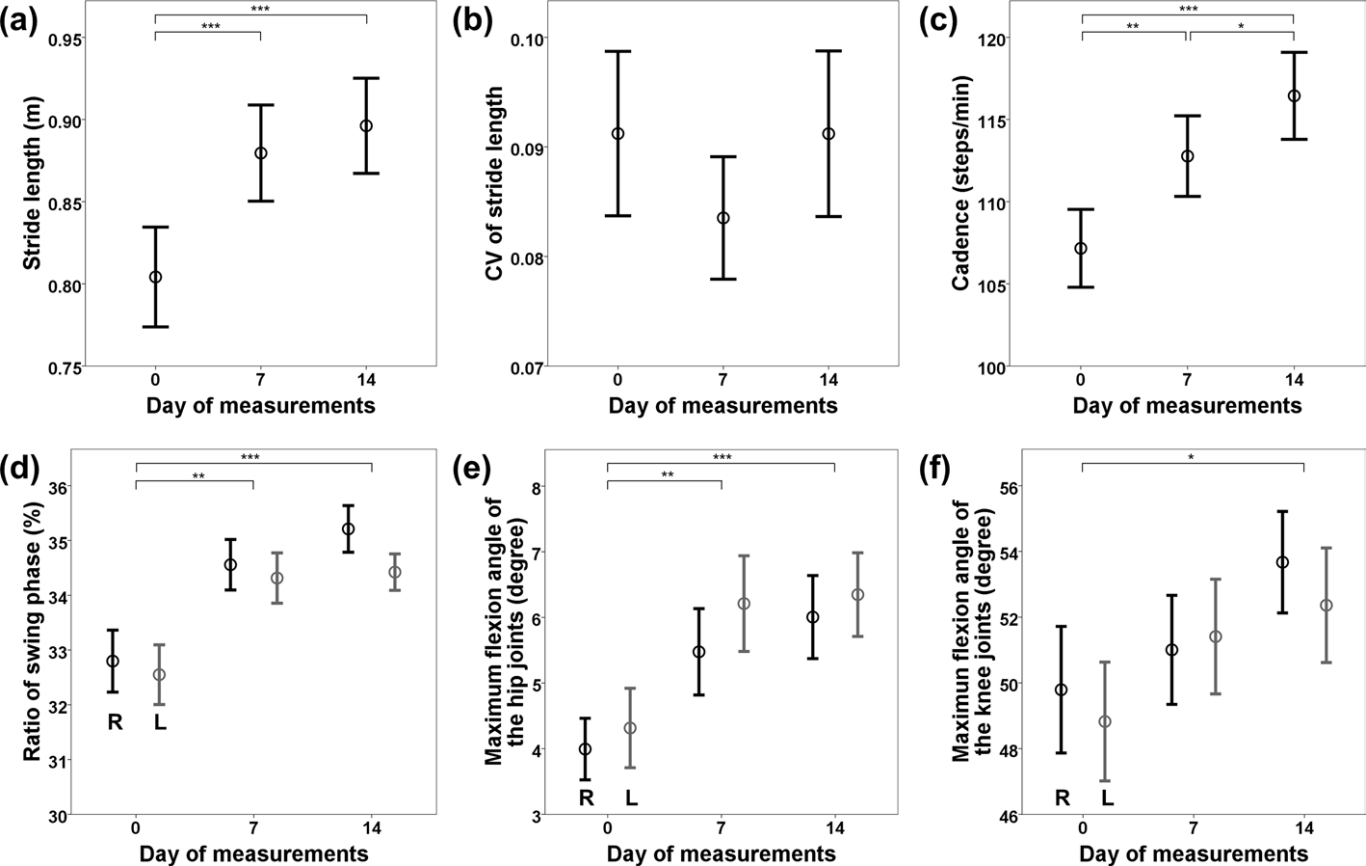
Distribution of gait parameters obtained by RehaGait®. Circles represent mean values and bars represent 95% confidence intervals. In panels **d**–**f**, black lines represent the value for the right lower limb (R) and gray lines for the left lower limb (L). **a** Stride length; **b** coefficient of variation (CV) of stride length; **c** cadence; **d** ratio of the swing phase; **e** maximum flexion angle of the hip joint, and **f** maximum flexion angle of the knee joint. *p < 0.05, **p < 0.01, ***p < 0.001

Figure 3 depicts the values of the gait parameters measured using RehaGait® (HASOMED, Magdeburg, Germany). All gait parameters, except for the SD of stride length (Fig. 3b) and duration, improved on day 14. There were statistically significant differences in stride length (Fig. 3a), cadence (Fig. 3c), ratio of the swing phase (Fig. 3d), and maximum flexion angles of the hip (Fig. 3e) and knee (Fig. 3f) between days 0 and 14.

## Discussion

Ataxic gait is characterized by reduced walking speed, cadence, stride length, and swing phase, as well as increased variability of stride length and duration, compared to the gait of healthy controls [18]. Therefore, it is reasonable to use these gait parameters to evaluate the efficacy of RAGT for people with ataxia. In the present study, all gait parameters, except variability, improved during the 12-day rehabilitation program using curara® for people with cerebellar ataxia. The improvements in gait parameters were considered to be responsible for the improvement in the 10 mWT and 6 mWD.

We excluded one participant from the analysis due to extreme outlier parameters. The improvement rates for 10 mWT and 6 mWD for this participant were lower than − 70%. This was an 84-year-old participant (the oldest in this study), with a BBS score of 27 (the lowest), SARA score of 17.5 (the second-highest), and 10 mWT of 36.2 s (the slowest) at baseline. We believe that this participant was not eligible for RAGT with curara®; however, she was highly motivated and completed the program with difficulty. There was considerable variability in the improvement rates of 10 mWT and 6 mWD among individuals. The 10 mWT of participants nos. 1 and 5 slightly worsened (14.0 and 7.9 s at baseline, respectively; 14.2 and 8.9 s post-intervention, respectively). In particular, the 10 mWT of participant no. 5 was the shortest, and was nearly equal to that of healthy controls. Therefore, the improvement rate of 10 mWT of participant no. 5 was relatively low. The 6 mWD of participant no. 10 did not improve (223 m at baseline and post-intervention). We considered the aforementioned slight deterioration or no change to be a normal variation, considering the differences in participant characteristics. Therefore, we did not exclude these results from the analysis.

We compared our results with those of two other randomized controlled trials [6, 8] (Table 2). The improvements in the 10 mWT observed in this study were comparable to those reported previously (19.0 ± 2.8% and 19.6 ± 4.1%, respectively) for a multidisciplinary, intensive, inpatient rehabilitation program of 28 days for Japanese people with cerebellar ataxia [6]. The SARA score did not change after the intervention in this study, while Miyai et al. reported a significant improvement in the total SARA score and truncal ataxia subscore after 4 weeks (i.e., at the end of the immediate intervention) [6]. There are several possible reasons for the discrepancy between gait parameters and SARA score in our study, as well as for the differences between the results of our study and those of Miyai et al. The rehabilitation program in our study exclusively focused on gait training, while that in Miyai et al. aimed to improve not only the ataxic gait, but also other ataxic symptoms, including speech disturbance and limb ataxia. However, we believe that the daily gait training in our study was more intensive compared to that in Miyai et al.

**Table 2:** Comparison of characteristics of the current study with those of previous studies

	Current study (*n* = 18)	Miyai et al. (immediate intervention group: *n* = 21) [6]	Bunn et al. (therapy group: *n* = 6) [8]
Intervention duration (weeks)	2	4	4
Intervention program	Robot-assisted gait training	Physical/occupational therapy	Home-based balance exercise
Age (years) Mean ± SD	62.8 ± 10.0	63.5 ± 11.0	60.2 ± 10.5
SARA score baseline Mean ± SD	10.6 ± 4.0	12.2 ± 3.2	11.8 ± 6.7
Difference in SARA score after the intervention Mean ± SD	− 0.1 ± 1.7	− 2.8 ± 1.8	− 1.8 ± 1.9
Improvement rate of 10 mWT (%) Mean	19.0	19.6	N/A

The improvement rate was calculated as the difference of values between post-intervention and pre-intervention divided by the value at pre-intervention. Each data in the study Miyai et al. were presented as “mean ± standard error”; thus, we calculated the standard deviation of each data in the study as follows: standard deviation = square root of sample size × standard error

N/A: not available; 10 mWT: 10-m walking time; SARA: Scale for the Assessment and Rating of Ataxia

The main outcome measures in this study were the improvement rates for the 10 mWT and 6 mWD; however, these parameters did not correlate with each other. The improvement rate for the 6 mWD, but not for the 10 mWT, correlated well with the SARA and BBS scores at baseline. Therefore, more severe cerebellar ataxia at baseline may predict a better outcome for the 6 mWD by RAGT with curara®. In other words, this may indicate a ceiling effect for the improvement in the 6 mWD, which may be because we evaluated the rate to determine efficacy, instead of the actual measurement value. Conversely, people with severe cerebellar ataxia cannot tolerate RAGT with curara®. Based on our preliminary screening results, we used a cut-off BBS score of 20 or greater as the inclusion criteria. However, further studies are required to determine the severity of ataxia that would benefit from RAGT with curara®.

The optimal methods and timing of therapist-assisted gait training for degenerative cerebellar ataxia are not standardized. Further, the mechanism underlying the temporary resolution of ataxic symptoms by intensive rehabilitation is not fully understood. One of the advantages of RAGT is that it provides a repetitive training program with certain assist conditions. As mentioned, extending the range of motion at the hip and knee joints and reducing the gait cycle was effective for gait training of people with cerebellar ataxia. Curara® can assist gait training under these conditions to stimulate the return to normal mobility for each person. Curara® currently does not automatically adjust the assist conditions; therefore, doctors or physical therapists are required to adjust the condition based on the gait parameters of the trainee. If techniques are developed for the automated assessment and condition adjustment in the future, curara® would be able to reduce the burden on doctors and therapists.

In this study, we have not assessed the neuromodulative effects of curara®; however, it may enable people with ataxia to perform gait training for a longer period of time by reducing gait-associated burden on the body. Therefore, repetitive, well-programmed RAGT may be a valid option for gait training for people with cerebellar ataxia.

## Study limitations

The major limitation of this study was that it was a single-arm study of 20 participants. Therefore, the extent of contribution of curara® to the study results cannot be determined. We did not use a non-weight-bearing device for gait training with curara®, so we exclusively enrolled participants who had the ability to walk for 30 min while wearing curara®. It is reasonable to assume that the ability to walk for 30 min under such a condition may itself be important for the favorable results. Further, we did not examine how long the effect lasts after RAGT with curara®. To address these issues, we plan to perform a randomized case–control study for people with cerebellar ataxia.

## Conclusions

Two-week RAGT with curara® and therapist-assisted training have comparable rehabilitative effects on gait function. Although the long-term effects after a month of RAGT with curara® are unclear, curara® is an effective tool for gait training of people with degenerative ataxia. We are enthusiastic to conduct a randomized controlled trial with curara® to verify its efficacy for the treatment of degenerative ataxia.

## Methods

### Participants and instruments

Twenty individuals (males: 8; females: 12) participated in this study. This study was registered in the Japan Registry of Clinical Trials (jRCTs032180164). The participants were recruited through the registry database. Participants were included if they had a definite diagnosis of SCA or possible or probable MSA-C using the second consensus criteria [19]; were aged ≥ 20 years; were able to walk ≥ 10 m independently, with or without a walker and/or brace; and had a BBS score of ≥ 20. Participants were excluded if they had a gait disturbance due to diseases other than SCA or MSA-C; did not fulfill the body-size criteria (underweight or obese) to fit into the curara®; or were otherwise inappropriate for the study (e.g., patients with severe dementia, psychiatric symptoms, severe spasticity, or leg joint contractures).

One participant had SCA type 2 (SCA2), 4 had SCA6, 3 had SCA31, and 1 had SCA36. Three participants had autosomal dominant cerebellar ataxia (without genetic testing), 3 had idiopathic cerebellar ataxia, and 5 had MSA-C. The mean age of participants at entry into the study was 63.5 ± 10.5 years (range: 45–84), BBS score was 40.1 ± 7.1, and SARA score was 11.3 ± 4.2.

**Fig. 4:**
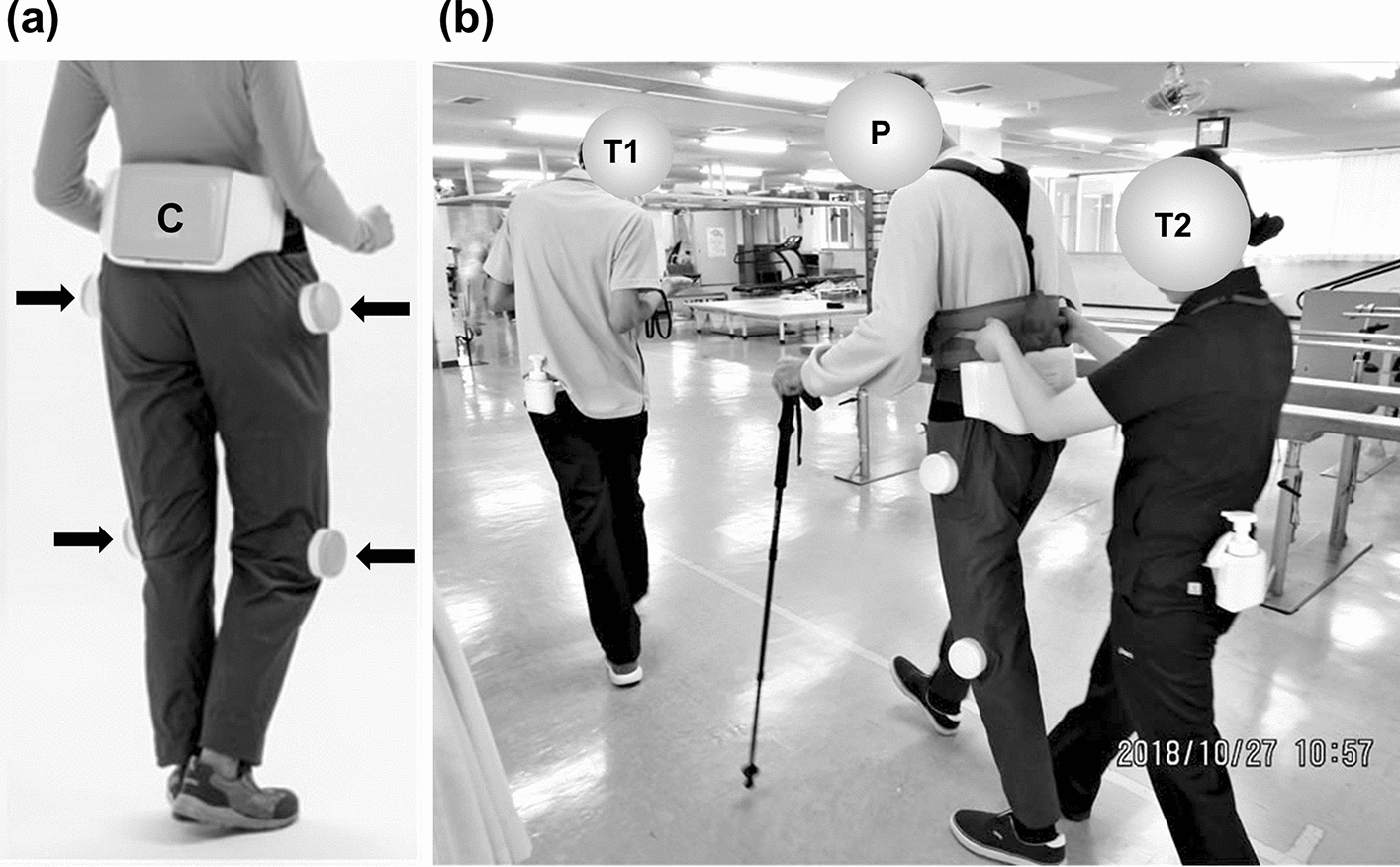
Appearance of the wearable curara® type 4 model (**a**) and gait training with curara® (**b**). **a** The controller box (C) and 4 actuator units (arrow) are indicated. **b** The participant (P) is accompanied by two physical therapists (T1 and T2) during gait training. Therapist T1 guides the participant and operates the mobile device to control curara®; T2 prevents the participant from falling

We used a wearable “curara® type 4” robot in this study (Fig. 4). The curara® type 4 weighs approximately 5 kg (4 actuator units and the controller box), and has an exoskeleton that does not have a direct connection to the hip and knee joints. The basic mechanisms of curara® are characterized by a torque-sensing technique and a synchronized-based control system. The actuator units move the hip and knee joints of the user, and are able to output a rated torque of about 7 Nm. The actuator consists of a motor and a gear; the power output of the motor is about 30 Watt. Curara® type 4 can assist both the flexion and extension of hip and knee joints during walking. The movement of joints by actuators directly increases the torques of hip and knee joint, which helps the user move their legs and walk forward. We have published the detailed information regarding curara® previously [16, 17]. Curara® is considered a pseudo-passive device [15] that consists of a streamlined structural design of small actuators and a small rechargeable battery.

### Measurements

The study duration was 15 days. We evaluated the participants at days 0, 7, and 14. On these days, they were instructed to walk 10 m on a flat floor at a comfortable speed 9 times, while wearing a RehaGait®. We measured the 10 mWT with a stopwatch. We collected the following gait parameters using RehaGait®: stride duration and length, SD of stride duration and length, cadence, ratio of the stance and swing phases, minimum and maximum knee joint angles, and minimum and maximum hip joint angles. We also measured the 6 mWD, SARA, and TUG tests at days 0, 7, and 14. The BBS score was evaluated at days 0 and 14. We selected the improvement rates of the 10 mWT and 6 mWD as the main outcome measures, calculated as the differences in values between days 14 and 0 divided by the value on day 0. All measurements mentioned above were made without wearing curara®.

### Rehabilitation program

All participants were instructed to perform gait training with curara® for 30 ± 5 min/day through days 2–6 and days 8–13 (total: 12 days). One or two physical therapists accompanied each participant to operate curara® and prevent falls (Fig. 4), but they did not provide the participants with any advice or suggestions. The participants also received a combination of physical (except gait training), occupational, and speech therapy rehabilitation. The maximum rehabilitation time, including gait training with curara®, was 3 h/day.

We set the synchronization gain, gait cycle, and joint angles as the assist conditions of curara®. Among these, synchronization gain was fixed to 0.1 at the hip joint and 0.3 at the knee joint throughout the rehabilitation period for all participants. The gait cycle and joint angles varied between individuals, and they were set according to the gait parameters of the fastest gait performance on day 0 or 7. The amplitude of the joint angle was set at 140% at the hip joint and 110% at the knee joint. The conditions set on day 0 were effective for gait training on days 1–6, and those set on day 7 were effective for days 8–13. These parameters were set such that the amount of assistance provided by the robot was greater at the hip joint compared to the knee joint, i.e., the device-in-charge robotic support was more influential at the hip joint compared to the knee joint. With these assist conditions, curara® supported the participants to reproduce their best gait performance during gait training.

### Statistical analysis

The differences in the SARA score, BBS score, and TUG test scores between days 0 and 14 were analyzed using a paired *t*-test. The differences in gait parameters obtained with RehaGait® were analyzed using a generalized linear mixed model with Bonferroni’s correction. In the model, the day of measurement was set as the fixed effect, and subject factors and the number of measurements on the same day of measurement were set as random effects. All statistical analyses were performed using SPSS Statistics software (version 24 for Windows; IBM Corp., Armonk, NY, USA). The level of significance was set at *p*-value < 0.05.

## Data Availability

The datasets used and/or analyzed during the current study are available from the corresponding author on reasonable request.

## Abbreviations

6 mWD: 6-min walking distance
10 mWT: 10-m walking time
BBS: Berg Balance Scale
MSA-C: Multiple system atrophy with predominant cerebellar ataxia
RAGT: Robot-assisted gait training
SARA: Scale for the Assessment and Rating of Ataxia
SCA: Spinocerebellar ataxia
TUG: Timed up and go
